# Continuous Lighting and High Daily Light Integral Enhance Yield and Quality of Mass-Produced Nasturtium (*Tropaeolum majus* L.) in Plant Factories

**DOI:** 10.3390/plants10061203

**Published:** 2021-06-12

**Authors:** Wenshuo Xu, Na Lu, Masao Kikuchi, Michiko Takagaki

**Affiliations:** 1 Graduate School of Horticulture, Chiba University, 648 Matsudo, Chiba 271-8510, Japan; xuwenshuo1988@163.com (W.X.); mtgaki@faculty.chiba-u.jp (M.T.); 2 Center for Environment, Health and Field Sciences, Chiba University, 6-2-1 Kashiwanoha, Kashiwa, Chiba 277-0882, Japan; m.kikuchi@faculty.chiba-u.jp

**Keywords:** continuous lighting, light stress, medicinal plant, RGR, vertical farming, PPFD, LUE, LAI

## Abstract

Nasturtium (*Tropaeolum majus* L.), as a medicinal plant, has a high phenolic content in its leaves and flowers. It is often used in salads as a dietary vegetable. Attracting strong demand, it could be a good candidate crop for a plant factory with artificial lighting (PFAL) that can achieve the mass production of high-quality crops with high productivity by regulating environmental conditions such as light. In this study, two experiments were conducted to investigate the effects of continuous lighting (CL) and different daily light integrals (DLIs) under CL on the growth, secondary metabolites, and light use efficiency (LUE) of nasturtium, all of which are essential in the successful cultivation in PFALs. In Experiment 1, two lighting models, the same DLI of 17.3 mol m^−2^ d^−1^ but different light periods (24 and 16 h) with different light intensities (200 and 300 µmol m^−2^ s^−1^, respectively), were applied to nasturtium. The results showed that leaf production, secondary metabolites, and LUE were higher under the 24-h CL treatment than under the 16-h non-CL treatment. In Experiment 2, three DLI levels (17.3, 25.9, and 34.6 mol m^−2^ d^−1^) under the CL condition were applied. The results showed that the growth parameters were positively correlated with the DLI levels under CL. The lowest DLI had the highest LUE. We conclude that the mass production of nasturtium under CL in PFALs is feasible, and the yield increases as DLI increases from 17.3 to 34.6 mol m^−2^ d^−1^ under CL without causing physiological stress on plants.

## 1. Introduction

Nasturtium (*Tropaeolum majus* L.) is a medicinal plant, and its leaves and flowers are rich in health-beneficial ingredients such as polyphenols and glucosinolates. The fresh leaves and flowers can be consumed in salads and sandwiches. Nasturtium as a functional herb, just like basil, coriander, and perilla, has great commercial prospects. The demand for nasturtium leaves and flowers increases rapidly, and the technology development for mass production in a plant factory has become particularly important [1].

In a plant factory with artificial lighting (PFAL), lighting control is one of the most important environmental control methods to regulate the production and quality of plants, such as controlling light periods, light intensities, or light spectra. The daily light integral (DLI) is the total amount of light received by plants in 24 h, which is an important index that combines both the light intensity and photoperiod, calculated by multiplying photosynthetic photon flux density (PPFD) by the light period. The DLI is generally proportional to the plant biomass within a certain range. For instance, a positive linear relationship between the DLI and leaf fresh weight (FW) of hydroponic lettuce was observed by increasing the DLI from 6.48 to 17.28 mol m^−2^ d^−1^, provided by LED lamps [2]. However, their results also indicated that the leaf FW of lettuce slightly decreased when the DLI increased from 14.40 to 17.28 mol m^−2^ d^−1^. A study conducted by Gao et al. [3] showed that the total shoot FW of hydroponic spinach decreased as the DLI increased from 17.3 to 20.2 mol m^−2^ d^−1^, provided by LED lamps. Yan et al. [4] showed that excessive DLI cannot improve the accumulation of a plant biomass. Besides, plants can acclimate to enhanced light radiation by increasing the level of secondary metabolites, including phenolic compounds [5]. The total phenolic contents in the shoot of a hydroponic coriander was significantly increased as the DLI increased from 5.76 to 17.28 mol m^−2^ d^−1^ under the root zone temperature of 20 ℃ [6]. We assume, therefore, that it is possible, by increasing the level of the DLI within a certain range, to increase the yield and bioactive components of nasturtium leaves.

The same DLI conditions can be generated either by a long light period with a low light intensity level or by a short light period with a high light intensity level. For the accumulation of a plant biomass, extending the photoperiod with a low light intensity level is more effective than increasing the light intensity with a short photoperiod under the same DLI [7]. For instance, under the same DLI of 8.64 mol m^−2^ d^−1^, the total FW and the total dry weight (DW) of hydroponic lettuce were heavier for the extended light period of 16 h than for the shorter light period of 12 h by 8.6% and 8.5%, respectively [8]. Weaver and van Iersel [9], who tested four different light periods (12, 15, 18, and 21 h) with the same DLI at 17 mol m^−2^ d^−1^ on lettuce grown in a greenhouse, found that the DW of lettuce was positively correlated with the photoperiod, and the highest DW was attained under 21 h. For lettuce and mizuna cultured under the DLI of 16 mol m^−2^ d^−1^ provided by white LED in a walk-in growth chamber, the shoot biomass of these crops grown under the photoperiod of 20 h was more than that of 10 h by 16.0% and 18.7%, respectively [10]. 

Continuous lighting (CL) is a lighting mode that maximizes the light period while minimizing the light intensity at the same DLI. The application of CL in a PFAL is considered an effective method to save the initial investment cost for lighting systems and operating costs for air conditioning and management [11,12,13]. The effects of CL, negative ones (such as leaf chlorosis, accelerated senescence, and development inhibition) on CL-sensitive plants, as well as positive ones (such as breeding acceleration, productivity increase, and quality improvement) on CL-tolerant plants, have both been widely recognized [7,14]. Although several hypotheses have been proposed, the precise mechanisms of these negative and positive effects have not been made clear yet. One of the hypotheses is that the negative effects on sensitive species may be due to the photooxidative damage caused by CL. Plant species with high antioxidant contents, therefore, could mitigate or avoid the injury caused by CL. For instance, under five different photoperiods (12, 14, 16, 20, and 24 h) with a PPFD of 200 µmol m^−2^ s^−1^ irradiated by blue LEDs, the highest antioxidant content (chlorogenic acid) of lettuce was obtained under the 24-h treatment, and it was explained that chlorogenic acid protected the plant body from photooxidative damage caused by CL [15]. Compared with 16 h, a greater shoot biomass and antioxidant content without leaf injury in hydroponic lettuce were obtained under 24 h at the same PPFD of 200 µmol m^−2^ s^−1^, provided by red and blue LEDs in a plant factory [16]. 

As a medicinal plant, nasturtium contains a higher antioxidant concentration than ordinary leafy vegetables, such as lettuce [17] and spinach [18]. We assume, therefore, that more plant biomass and the accumulation of bioactive compounds of nasturtium plants can be achieved by applying CL, compared with shorter lighting durations under the same DLI. The LED lighting is ideal in controlling the light environment for the purpose of increasing the yield and nutrient contents of crops. However, the electricity cost the lighting entails accounts for 70–80% of the total electricity consumption of the crop production in PFALs [19] (pp. 7–33). It is necessary to improve concomitantly the light use efficiency (LUE) by adopting appropriate light environment management methods. The purpose of this study was to investigate the effects of CL and different DLIs on the growth of nasturtium, its nutrient contents, and the LUE in growing the plant, with a view toward providing indispensable information for the effective management of light conditions for nasturtium production in PFALs.

## 2. Materials and Methods

### 2.1. Treatments and Growth Conditions

Two experiments were conducted in this study.

**Figure 1:**
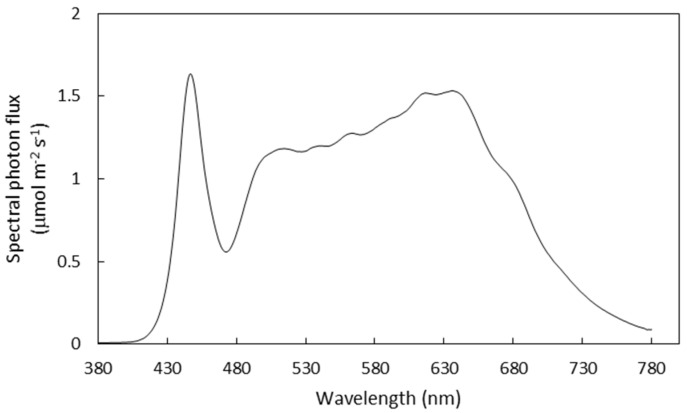
Spectral distribution of the LED lamp used in the experiments.

In Experiment 1, seedlings were propagated by cutting propagation. The apical buds taken from 50-day-old parental plants were used as cuttings. Thirty-two cuttings were planted in a hydroponic container (L × W × H: 65 × 42 × 10 cm^3^) and filled with 20-L nutrient solution (Otsuka hydroponic composition, as described by Xu et al. 2021) for rooting. Air pumps were used to pump air into the nutrient solution which was adjusted to the electrical conductivity (EC) at 1.0 ± 0.1 dS m^−1^ and pH at 6.5 ± 0.1. The light intensity and light period were 200 µmol m^−2^ s^−1^ in PPFD and 14 h per day, respectively. The light was provided by white LED lamps (Suri-g, Otsuka, Japan; the light spectrum is shown in Figure 1). The light intensity was measured at the surface of the cultivation panels using a light meter (LI 250A, LI-190R; Li-Cor Inc., Lincoln, NE, USA).

Two weeks after planting, uniform seedlings were transplanted to a deep-flow technique hydroponic system, adjusting the nutrient solution to EC at 2.0 ± 0.1 dS m^−1^ and pH at 6.5 ± 0.1. The initial planting density was 21.5 plants m^−2^. Two treatments were performed on the seedlings: a 16-h light period under the PPFD of 300 µmol m^−2^ s^−1^ and 24-h light period under the PPFD of 200 µmol m^−2^ s^−1^ (hereafter: T16-300 and T24-200, respectively) at the same DLI level of 17.3 mol m^−2^ d^−1^ for 3 weeks (Table 1). The LED lamps and the measurements of the light intensity were the same as described for the cutting propagation. During the entire cultivation period, plants were grown under an ambient CO_2_ concentration. The air temperature and relative humidity (RH%) were maintained at 21 °C and 50–80%, respectively. Plant spacing was adjusted for at 7 and 14 days after treatment (DAT) to make the plant density during the 1st, 2nd, and 3rd weeks 21.5, 16.7, and 11.1 plants m^−2^, respectively.

In Experiment 2, uniform seedlings, propagated in the same way as in Experiment 1, were transplanted two weeks after planting to the same hydroponic system as in Experiment 1, with an initial planting density of 16.7 plants m^−2^. The seedlings were grown under continuous lighting (CL) throughout a three-week period with 3 different DLI treatments: 17.3, 25.9, and 34.6 mol m^−2^ d^−1^ (Table 1). These three DLI treatments were created by three different PPFD levels: 200, 300, and 400 µmol m^−2^ s^−1^ (denoted hereafter as CL-200, CL-300, and CL-400, respectively). The LED lamps and light intensity measurements were the same as in Experiment 1. During the entire cultivation period, all plants were grown under the same CO_2_, air temperature, and RH% conditions as in Experiment 1. Plant spacing was adjusted for at 7 and 14 DAT to make the plant density during the 1st, 2nd, and 3rd weeks 16.7, 11.1, and 5.6 plants m^−2^, respectively. This change in plant density was based on the results of Experiment 1. This issue shall be discussed later in the Discussion section.

### 2.2. Measurement

#### 2.2.1. Growth Parameters

In Experiment 1, at 7, 14, and 21 DAT, six plants were sampled in each treatment to evaluate the growth properties. The total shoot FW, leaf FW, root FW, leaf area, and leaf number were measured. The shoot and root samples were placed in an oven (80 °C) for 1 week to determine the shoot dry weight (DW), leaf DW, and root DW. The leaf area per plant (m^2^/plant) was determined by using a Li-3000 leaf area meter (Li-Cor, Lincoln, NE, USA). In Experiment 2, at 7, 14, and 21 DAT, eight plants were sampled in each treatment to measure the same eight growth parameters in the same manner as for Experiment 1. 

The relative growth rate (RGR) was calculated from the shoot DW as RGR = [ln(W_i_)-ln(W_0_)]/(i-0), where W_i_ and W_0_ are the DW of the shoot biomass of nasturtium at i days after the treatment and at the start of the treatment, respectively. The leaf area index (LAI, m^2^/m^2^) was calculated as LAI = leaf area per plant × number of plants per m^2^. The relationship between the RGR and LAI was examined by means of the simple regression analysis.

#### 2.2.2. Chlorophyll Fluorescence Characteristics

Chlorophyll fluorescence parameters were measured with an open gas exchange system (LI-6400XT, Li-Cor, Inc., Lincoln, NE, USA) and an integrated fluorescence chamber head (LI-6400-40XT, Li-Cor, Inc., Lincoln, NE, USA). 

In Experiment 1, the chlorophyll fluorescence parameters of nasturtium leaves from six plants per treatment were measured at 21 DAT. After darkening the plants for 30 min, using a leaf clip on the leaf, the minimal fluorescence in the dark-adapted state (F_min_) was recorded, and a saturating pulse of radiation (>7000 μmol m^−2^ s^−1^) was given for 0.8 s to determine the maximal fluorescence in the dark-adapted state (F_max_). The maximum quantum yield of the PSII primary photochemistry (F_v_/F_max_) was calculated as (F_max_−F_min_)/F_max_. The F_v_/F_max_ is a sensitive stress indicator; if the value of this ratio is equal to or higher than 0.8 for a plant, it indicates that the plant is not suffering from light stress [20,21].

In Experiment 2, the values of F_min_ and F_max_ of the dark-adapted (30 min) fully expanded leaves from eight plants per treatment were measured at 6 and 20 DAT. The F_v_/F_max_ was calculated in the same way as in Experiment 1. After determining the F_min_ and F_max_, the same leaves were illuminated with an actinic light for 30 min to record the steady-state fluorescence in the light (F_s_). For leaves treated under CL-200, CL-300, and CL-400, the actinic light intensity was set up at 200, 300, and 400, respectively. Following this irradiation, a saturation flash (>7000 μmol m^−2^ s^−1^) was given for 0.8 s to determine the maximal fluorescence yields in the light-adapted state (F_max_’). After the flash, the actinic radiation was turned off, and a far-red radiation was given to record the minimal fluorescence yield in the light (F_min_’).

The effective quantum yield of PSII (PhiPSII) and the coefficient of photochemical quenching (qP) and nonphotochemical quenching (qN) [22] were calculated as follows:

PhiPSII = (F_max_’ − F_s_)/F_max_’

qP = (F_max_’− F_s_)/(F_max_’− F_min_’)

qN = (F_max_− F_max_’)/(F_max_− F_min_^’^)

#### 2.2.3. Antioxidant Activity (1,1-Diphenyl-2-picrylhydrazyl Assay)

At 7, 14, and 21 DAT, the plant leaves from each treatment were sampled and stored at −60 °C for the subsequent analyses (*n* = 6 in Experiment 1 and *n* = 8 in Experiment 2). 

A frozen leaf sample, 0.5 g each, was homogenized with 5 mL of 80% (*v/v*) methanol in an ice bath for 1 min. The homogenate was centrifuged at 4 °C at 10,000× *g* for 30 min. The supernatant was transferred, together with 80% methanol, to a graduated cylinder to make up 6 mL of solution. A well-mixed solution of 1.5 mL was transferred to an EP tube and then stored at −20 °C until further analysis.

The spectrophotometric analysis, described by Nguyen et al. [6], was used to determine the scavenging activity of the 1,1-diphenyl-2-picrydrazyl (DPPH) radicals of the nasturtium leaves. Each sample extract of 50 µL and the standard (Trolox) were added to 2 mL of methanol DPPH solution (80 µM). Each of the mixtures was incubated in the dark for 30 min at room temperature. The absorbance of each mixture was read at 517 nm using a spectrophotometer (ASV11D, As One, Corp., Osaka, Japan). The standard curve was prepared with different concentrations of Trolox solution (0, 100, 200, 300, 400, 500, 600, 700, 800, and 900 µM). The Trolox standard curve equation (R^2^ = 0.99) was used to determine the DPPH radical-scavenging activity of each sample solution. The results were expressed as milligrams of Trolox equivalent (TE) per gram of FW (mg TE/g FW).

#### 2.2.4. Total Phenolic Content

The leaf extract was prepared as described in Section 2.2.3. The total phenolic content (TPC) of the nasturtium leaf was determined using the Folin–Ciocalteu colorimetric assay described by Nguyen et al. [6]. Each sample extract of 0.25 mL and the standard (gallic acid) was added to 1.25 mL of 10% Folin–Ciocalteu reagent to be neutralized by 1 mL of 7.5% sodium carbonate solution. Each of the mixtures was incubated for 1 h at room temperature. The absorbance of each mixture was measured at 765 nm using a spectrophotometer. The standard curve was prepared with different concentrations of gallic acid (0, 0.05, 0.075, 0.1, 0.125, 0.15, 0.175, 0.2, 0.25, and 0.3 mg/mL). The gallic acid standard curve equation (R^2^ = 0.99) was used to determine the TPC of each sample solution. The results were expressed as milligrams of gallic acid equivalents (GAE) per gram of FW (mg GAE/g FW).

#### 2.2.5. Light Use Efficiency

The light use efficiency (LUE) was determined as follows: LUE = d/PAR, where d is the rate of increase of the nasturtium leaf dry mass per plant (g m^−2^ h^−1^), and PAR is the photosynthetically active radiation received at the plant community surface (MJ m^−2^ h^−1^). In Experiment 1, the PAR was the same for the two lighting treatments: T16-300 and T24-200: 0.1519 MJ m^−2^ h^−1^. In Experiment 2, the PAR was 0.1519, 0.2278, and 0.3038 MJ m^−2^ h^−1^ for CL-200, CL-300, and CL-400, respectively. The energy of the photons was calculated according to Planck’s equation based on the light spectrum. The energy of 1-mol photons was calculated as E = L·h·c/λ, where L is the number of 1-mol photons (6.02 × 10^23^), h is the Planck constant (6.6262 × 10^−34^ J s), c is the speed of light (2.9979 × 10^8^ m s^−1^), and λ is the wavelength.

#### 2.2.6. Statistical Analyses

ANOVA, two-group mean comparison (by *t*-test), multiple mean comparison (by Tukey’s method), and linear regression were the statistical analyses applied for the data obtained from the two experiments conducted in this study. The critical significance level of *p* = 0.05 was adopted for the statistical tests in these analyses. Whenever possible, the probability of the test statistics was made explicit numerically or by using the following signs: ^1^*, ^2^*, ^3^*, ^4^*, ^5^*, …, indicating *p* < 0.05, *p* < 0.01, *p* < 0.001, *p* < 1.0 × 10^−4^, *p* < 1.0 × 10^−5^, …, respectively. SPSS statistical software (IBM SPSS Statistics, Version 19.0. Armonk, NY, USA: IBM Corp.) was used for all the statistical analyses in this study.

## 3. Results

### 3.1. Plant Growth in Experiment 1

**Figure 2:**
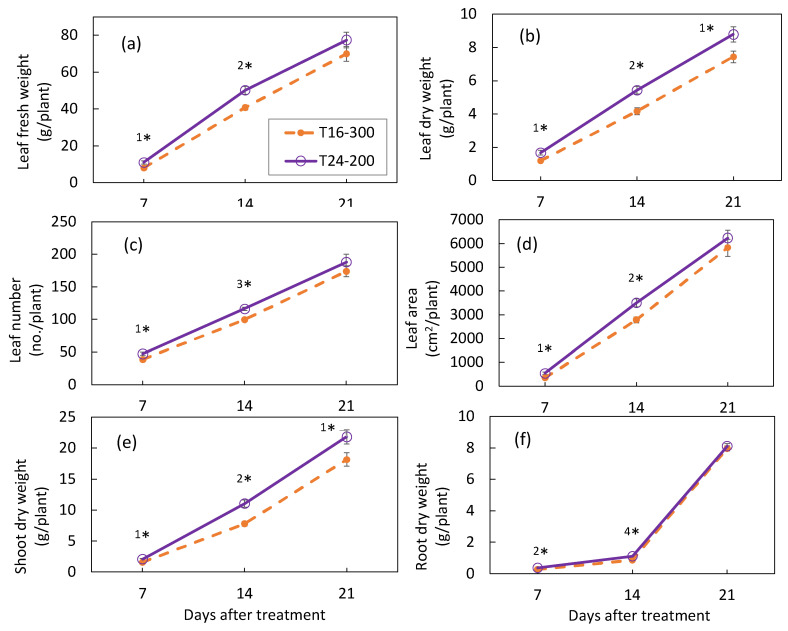
Leaf fresh (**a**) and dry weight (**b**), leaf numbers (**c**), leaf area (**d**), shoot dry weight (**e**), and root dry weight (**f**) of nasturtium grown under T16-300 and T24-200 at 7, 14, and 21 days after treatment. Values are the means ± SE (*n* = 6). Asterisks indicate significant differences between the treatments (^1^*, *p* < 0.05; ^2^*, *p* < 0.01; ^3^*, *p* < 0.001, and ^4^*, *p* < 0.0001), determined by the *t*-test. Plant density during the 1st, 2nd, and 3rd weeks was 21.5, 16.7, and 11.1 plants m^−2^, respectively.

The growth characteristics of nasturtium plants grown under different light periods in Experiment 1 are summarized in Figure 2. The biomass—above-ground, as well as root—increased as the plant growth progressed, though the growth pattern was contrasted between the above-ground and the root. The growth rate of the above-ground biomass increased almost linearly during the cultivation period (Figure 2a–e), whereas that of the root accelerated from the second week to the third week (Figure 2f). More importantly, during the earlier growth stages of the first and the second weeks, the mean levels of the growth parameters were unanimously higher for T24-200 than for T16-300, with all the mean differences that were statistically significant, while, at the end of the third week, there was no significant mean differences found for all the growth parameters, except for the dry masses of the leaves and shoots. Even for these two growth parameters, the rate of the difference between T16-300 and T24-200 decreased substantially, from 30–40% at 7 DAT to 18–20% at 21 DAT (Figure 2 and Supplementary Table S1 for the numerical data).

**Figure 3:**
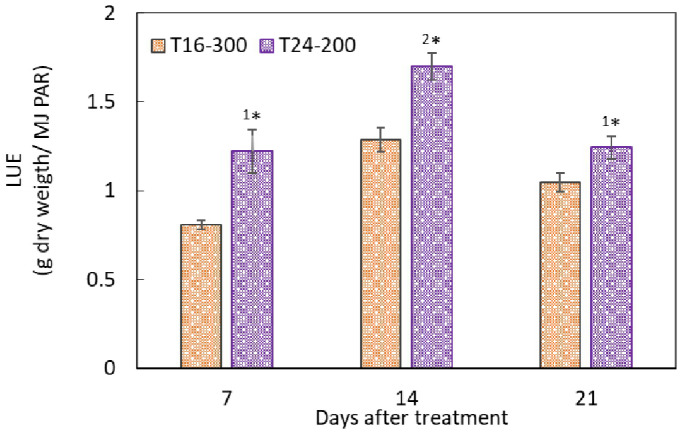
Light use efficiencies (LUE) in the leaf dry masses of nasturtium grown under T16-300 and T24-200 measured at 7, 14, and 21 days after treatment. Values are the means ± SE (*n* = 6). The asterisk sign indicates significant differences between the treatments (^1^*, *p* < 0.05 and ^2^*, *p* < 0.01), determined by the *t*-test.

The LUE of the nasturtium cultivation was significantly higher under T24 than under T16 at 7, 14, and 21 DAT (Figure 3).

### 3.2. Light Stress Indicator, Antioxidant Capacity, and Total Phenolic Content in the Leaves of Nasturtium in Experiment 1

Although the F_v_/F_max_ of nasturtium in Experiment 1 was significantly higher under T16-300 than under T24-200 at 14 and 21 DAT (Table 2), the levels of this yield were over 0.8 for both light period treatments and for both growth stages. This means that there was no light stress for the leaves under continuous lighting [20,21]. 

For each light period treatment, the antioxidant capacity of nasturtium decreased significantly from the second to the third growth stage, and for each growth stage, it was significantly higher under T24 than under T16 (Table 2). For each light period treatment, the TPC tended to increase, but no difference was statistically detected between the second and the third growth stages. For the second and third growth stages, the TPC tended to be higher under T24 than under T16, but that difference was statistically significant only at the second growth stage, not at the third growth stage.

For each light period treatment, the RGR of nasturtium decreased significantly from the first and second to the third growth stage, and for each growth stage, it was significantly higher under T24 than under T16 (Table 2). For each light period treatment, the LAI of nasturtium increased significantly from the first to the second and from the second to the third growth stage. For the earlier growth stages, the LAI was significantly higher under T24 than under T16, but the difference disappeared by the end of the third growth stage. 

### 3.3. Plant Growth in Experiment 2

**Figure 4:**
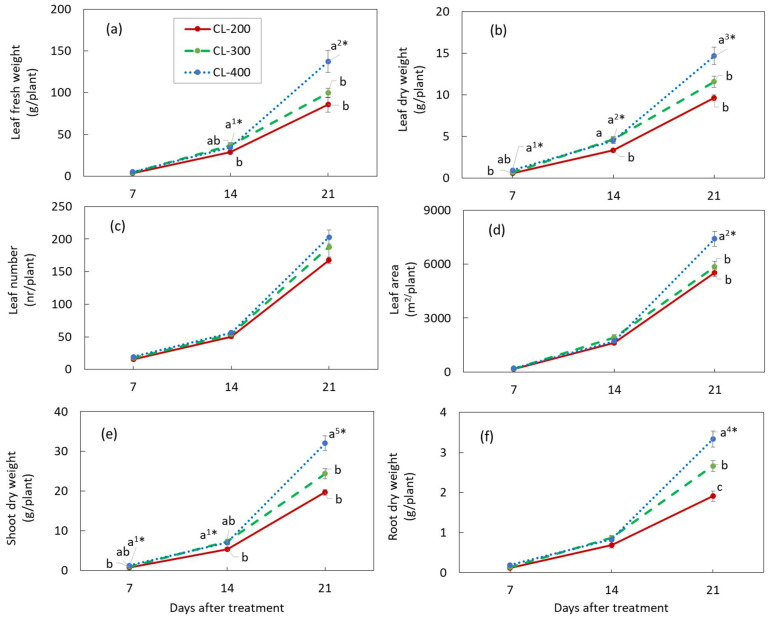
Leaf fresh (**a**) and dry weight (**b**), leaf numbers (**c**), leaf area (**d**), shoot dry weight (**e**), and root dry weight (**f**) of nasturtium grown under CL-200, CL-300, and CL-400 at 7, 14, and 21 days after treatment. CL-200, CL-300, and CL-400 represent the light period of 24 h per day with a light intensity at 200, 300, and 400 µmol m^−2^ s^−1^, respectively. Values are the means ± SE (*n* = 8). Different alphabet letters indicate significant differences between the treatments, the signs, ^1^*, ^2^*, ^3^*, ^4^*, or ^5^* put after the alphabet ‘a’ at the largest mean for each parameter and each ‘days after treatment’, indicating that the probability of the ANOVA (the multiple mean comparison by Tukey) was *p* < 0.05, *p* < 0.01, *p* < 0.001, *p* < 1.0 × 10^−4^, or *p* < 1.0 × 10^−5^, respectively. The plant density during the 1st, 2nd, and 3rd weeks was 16.7, 11.1, and 5.6 plants m^−2^, respectively.

**Figure 5:**
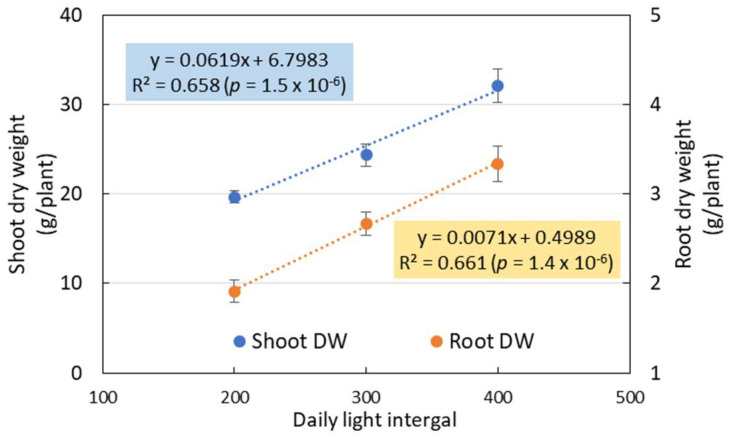
Relationship between the biomass (shoot and root dry weight) of the nasturtium plant (y) and the daily light integral (DLI; in terms of the light intensity) (x) at the last stage of plant growth (21 DAT). Each dot shows the mean ± SE (*n* = 8), and the R^2^ and the probability of the regression estimation are estimated using the raw data (*n* = 24).

The growth characteristics of nasturtium plants grown under different light intensities in Experiment 2 are summarized in Figure 4 (Supplementary Table S2 for the numerical data). The growth parameters measured in this experiment altogether discerned a pattern of plant growth for the growth period of three weeks after planting: The plants grew better under higher light intensities than under lower ones. At the earlier growth stage of the first week after planting, no significant mean differences were found for all the growth parameters, except for the leaf DW (Figure 4b) and the shoot DW (Figure 4e), similar to the second week after planting, except in addition to the leaf FW (Figure 4a). At the end of the third week, the mean levels of the growth parameters were unanimously higher for CL-400 than for CL-300 and CL-200, with all the mean differences that were statistically significant, except for the leaf number. Additionally clear at the end of the third growth stage was a highly significant linear relationship between the DLI and the biomass of nasturtium plants (Figure 5). For both the shoot and root, the DW increased linearly as the DLI, expressed by the light intensity, increased.

**Figure 6:**
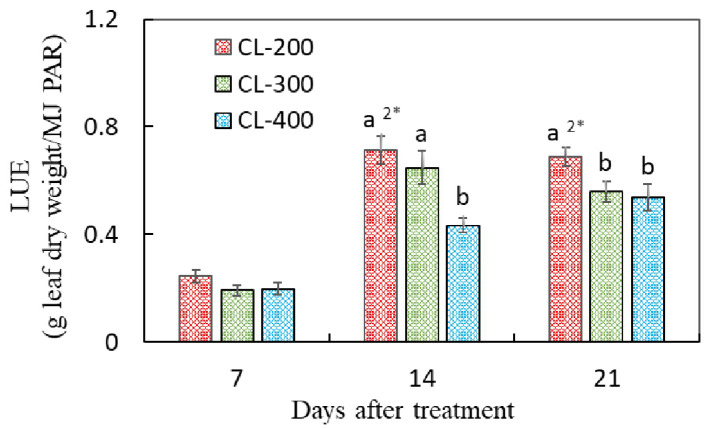
Light use efficiencies (LUE) of nasturtium grown under CL-200, CL-300, and CL-400 at 7, 14, and 21 days after treatment. Values are the means ± SE (*n* = 8). Different alphabet letters indicate significant differences between the treatments, the signs ^2^* put after the alphabet ‘a’ at the largest mean for each ‘days after treatment’, indicating that the probability of the ANOVA (the multiple mean comparison by Tukey) was *p* < 0.01.

Contrasting with the biomass, the LUE of the nasturtium cultivation was significantly higher under CL-200 than under CL-300 and CL-400 at 21 DAT (Figure 6). 

### 3.4. Antioxidant Capacity and Total Phenolic Content in the Leaves of Nasturtium in Experiment 2

Changes in the light intensity under continuous lighting did not give any significant changes in the antioxidant capacity and total phenolic content of nasturtium throughout the three growth stages (Table 3). 

For each light intensity treatment, the RGR of nasturtium decreased significantly from the first and the second to the third growth stage, and for each growth stage, significant differences were observed among the treatments (Table 3). For each light intensity treatment, the LAI of nasturtium increased significantly from the first to the second and, further, to the third growth stage. For each growth stage, the mean levels of the LAI were significantly different only at the third growth stage but not at the first and second growth stages.

### 3.5. Chlorophyll Fluorescence Characteristics of Nasturtium in Experiment 2

**Figure 7:**
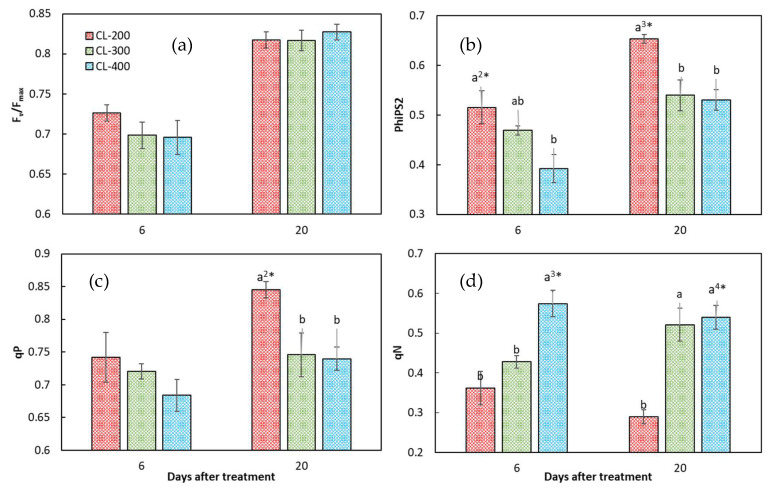
The maximum quantum yield of the PSII primary photochemistry (F_v_/F_max_) (**a**), quantum yield of the PSII electron transport (PhiPS2) (**b**), and the coefficients of photochemical quenching (qP) (**c**) and nonphotochemical quenching (qN) (**d**) of nasturtium grown under CL-200, CL-300, and CL-400 at 6 and 20 days after treatment in Experiment 2. CL-200, CL-300, and CL-400 represent the light period of 24 h per day with a light intensity at 200, 300, and 400 µmol m^-2^ s^−1^, respectively. Values are the means ± SE (*n* = 8). Different alphabet letters indicate significant differences between the treatments, the signs, ^2^*, ^3^*, or ^4^* put after the alphabet ‘a’ at the largest mean for each parameter and each ‘days after treatment’, indicating that the probability of the ANOVA (the multiple mean comparison by Tukey) was *p* < 0.01, *p* < 0.001, or *p* < 1.0 × 10^−4^, respectively.

No significant differences among the light intensity treatments were observed for the F_v_/F_max_ (Figure 7a). Except for this parameter, for the other parameters related to the chlorophyll fluorescence characteristics, two different patterns of response to the increase in light intensity were clearly discerned. One was a positive response, in that the parameters increased as light intensity increased, and the other was a negative response, in that the parameters decreased as the light intensity increased. Nonphotochemical quenching (qN) belonged to the positive response group (Figure 7d), and the quantum yield of the PSII electron transport (PhiPS2) and the coefficient of photochemical quenching (qP) belonged to the negative response group (Figure 7b,c). 

It should be noted that, as far as the means were concerned, the parameters of those in the positive response group increased, and those in the negative response group decreased, from CL-200 to CL-400, regardless of the growth stage. There were cases in which the mean differences between CL-200 and CL-300 or between CL-300 and CL-400 were not statistically significant. If the comparison was between CL-200 and CL-400, however, the mean differences were all statistically significant, with the only exception for qP at 6 DAT.

### 3.6. Relationship between RGR and LAI in Experiment 1 and Experiment 2

Both Experiment 1 and Experiment 2 revealed a clear linear relationship between the RGR and LAI (Supplementary Figure S1). For both experiments, the slope coefficient of the regression line decreased as the growth stage progressed, but at the same growth stage, the differences in the slope coefficients between or among the lighting treatments tended to not be large (statistically verified, though the results are not shown). With these variations, however, the linearity of the LAI–RGR relationship was significant for all the cases.

## 4. Discussion

### 4.1. Effects of CL on the Growth and LUE of Hydroponic Nasturtium under the Same DLI

In Experiment 1, under the same DLI (17.28 mol m^−2^ d^−1^), the dry mass of the nasturtium plant was significantly enhanced by extending the light period from 16 to 24 h, rather than increasing the light intensity from 200 to 300 µmol m^−2^ s^−1^ (Figure 2). A similar trend has been reported in several leafy vegetables, such as lettuce and mizuna [8,9,10]. It is reported that, under the same DLI, the plant growth could be better with a longer photoperiod and lower PPFDs, because the photosynthetic efficiency would be driven higher under such a lighting environment than otherwise [9]. It is also reported that CL usually causes leaf damage in CL-sensitive plants [23,24]. This study found that nasturtium grown under CL (T24-200) attained a heavier plant biomass and faster RGR compared to the plant grown under 16 h (T16-300) (Figure 2 and Table 2), without causing any physiological leaf injuries (F_v_/F_max_ > 0.8; Table 2). Nasturtium could be a CL-tolerant crop, which makes it an ideal candidate crop for PFAL production. The LUE of nasturtium was significantly higher under T24-200 than under T16-300 for all the growth stages tested (Figure 3). CL can therefore effectively promote the production of nasturtium in PFALs while improving the LUE. CL is a feasible technology in PFAL production to increase the nasturtium yield, as well as to enhance the energy efficiency.

### 4.2. Effects of CL on the Growth and LUE of Hydroponic Nasturtium under Different DLIs

Experiment 2 showed that the growth characteristics of hydroponic nasturtium were significantly affected by the DLI (Figure 4). The biomass of nasturtium was significantly increased by increasing the DLI from 17.3 to 34.6 mol m^−2^ d^−1^ under CL. We found that the positive linear relationships between the shoot and root biomass and DLI were all highly significant at the last stage of plant growth (Figure 5). 

Such linear relationships were obtained between the fresh and dry mass of shoots of green butterhead lettuce and the DLI from 6.9 to 15.6 mol m^−2^ d^−1^, provided by LED lamps (R^2^ = 0.89 and 0.85, respectively) [25]. Dou et al. [26] found such linear relationships for the shoot FW and DW of sweet basil with a DLI from 9.3 to 17.8 mol m^−2^ d^−1^ (R^2^ = 0.79 and 0.77, respectively), though the highest shoot FW and DW were obtained at 16.5 mol m^−2^ d^−1^ instead of 17.8 mol m^−2^ d^−1^. Their study, together with other studies [2,3], indicated that the DLI displays positive effects on the plant growth within a certain range, beyond which range an excessive DLI could inhibit the plant growth. In the present study, however, the nasturtium biomass increased for the DLI range from 17.3 to 34.6 mol m^−2^ d^−1^, and the highest biomass of the shoots, leaves, and roots was obtained at 34.6 mol m^−2^ d^−1^ (Figure 4 and Figure 5). Compared with leafy vegetables, such as lettuce or basil, nasturtium seemed to have more tolerance not only for a longer light period, as found in Experiment 1, but, also, for much higher amounts of light, as found in Experiment 2. 

These results indicate that there is still room to raise the DLI for enhancing the yield of nasturtium grown under CL. A further study is needed to identify the level of DLI beyond when the DLI becomes a growth inhibitive factor. The LUE of nasturtium was significantly higher under lower DLIs (17.3 and 25.9 mol m^−2^ d^−1^ for 14 DAT and 17.3 mol m^−2^ d^−1^ for 21 DAT) than under higher DLIs (34.9 mol m^−2^ d^−1^ for 14 and 21 DAT) (Figure 6). It is suggested that a lower DLI could increase the LUE of nasturtium, especially during the later growth stage of the plant. These results could be explained by qN, which was significantly lower under the lower DLI than that under the higher ones at 20 DAT (Figure 7d). Compared with the lower DLIs (CL-200), nasturtium under higher DLIs (CL-300 and CL-400) dissipated most of the absorbed light energy in the form of heat rather than use it for photochemistry.

### 4.3. Effects of CL on the Tolerance of Hydroponic Nasturtium under the Same and Different DLIs

As we explained before, CL, not to mention CL with a high light intensity, could induce adverse effects on plants caused by excess light energy. The chlorophyll fluorescence parameters can reflect the light absorption, transfer, dissipation, and distribution abilities in the photosystem II (PS II) of plants. Therefore, these parameters are the most reliable and widely used indexes to evaluate the photosynthesis ability and abiotic stress response of plants [27]. 

In Experiment 1, the values of F_v_/F_max_ of nasturtium grown under T24-200, as well as under T16-300, were higher than 0.8 at 21 DAT (Table 2), which indicated that the nasturtium plants were not subjected to light stress under CL in Experiment 1. 

A reversible photo inhibition caused by light stress was observed in Experiment 2 (Figure 7). At 6 DAT, the values of F_v_/F_max_ of the nasturtium plants under all treatments were lower than 0.8, indicating that plants were suffering from light stress. At this early growth stage, the F_v_/F_max_ and PhiPS2 decreased as the light intensity increased from 200 to 400, suggesting a greater degree of photoinhibition for higher light intensities. The decline in the F_v_/F_max_ was indicative of photodamaged PSII due to a decrease in the proportion of open PSII reaction centers (qP). Additionally, the decrease in PhiPS2 could be attributed to both the decrease in the proportion of open PSII reaction centers (lower qP) and the increase in light energy dissipated in the form of heat (larger qN).

At 20 DAT, an elevation of the F_v_/F_max_ to over 0.8 for all the DLI treatments and the diminished among-treatment differences for the F_v_/F_max_ were observed, which implies that the photoinhibition was recovered at 20 DAT. The elevation of the F_v_/F_max_ could be explained by the increase in qP, which allowed for more absorbed energy to be used for photochemistry rather than dissipated in the form of heat (qN). As a result, the quantum yield of the PSII electron transport (PhiPS2) was improved. The diminished differences in the F_v_/F_max_ among the treatments could be explained by the lager differences in the qP and qN. The levels of qP under CL-300 and CL-400 were lower than those under CL-200, whereas it was symmetrically the other way around for the levels of qN (Figure 7). These results indicated that, under high light intensities, the photosynthetic apparatus was protected from light stress by the greater ability of the nasturtium plants to dissipate the excess excitation energy as heat. 

A similar reversible photo inhibition case was observed in lettuce, where 25-day-old lettuce plants were treated under CL with three different light intensities (100, 200, and 300 µmol m^−2^ s^−1^) for 12 days [28]. In this case, the lettuce plants were suffering from photoinhibition under 200 and 300 µmol m^−2^ s^−1^ at 6 DAT, demonstrated by the lower values of the F_v_/F_max_, PhiPS2, and qP and the higher value of qN compared to the values under 100 µmol m^−2^ s^−1^, and the plant recovered from this photoinhibition at 12 DAT, as manifested by the increases in the F_v_/F_max_, PhiPS2, and qP.

### 4.4. Effects of DLI and CL on Antioxidants of Hydroponic Nasturtium

Plants can acclimate to enhanced light radiation by improving their antioxidant capacity [5]. This has been well-described in several crops, such as sweet potatoes [29] and lettuce [30].

In Experiment 1, compared with the 16-h light period, the TPC in nasturtium leaves was higher under CL at 14 DAT, but there was no significant difference at 21 DAT, and the antioxidant capacity was higher at both 14 and 21 DAT under CL (Table 2). A study reported that CL continuously increased the accumulation of carbohydrate and nicotinamide adenine dinucleotide hydrogen phosphate (NADPH) in plant leaves. An excessive NADPH accumulation in the chloroplast stroma eventually induces the synthesis of a superoxide anion radical (O_2_^−^). In order to protect the photosynthetic apparatus against oxygen damage, plants synthesize antioxidants (such as phenolic compounds) to scavenge O_2_^−^ [28]. 

In Experiment 2, the levels of antioxidant capacity and TPC were higher at the earlier growth stage (week 1) than at the later growth stages (weeks 2 and 3), whereas there were no significant differences among the different DLI treatments at the same growth stage (Table 3). The former observation could be understood, as the young seedlings at the first growth stage need time to acclimate to CL. The latter observation suggests that CL under these levels of DLI does not create any extra harmful stress on the growth of nasturtium plants. 

Thus, CL, compared with shorter light periods, could be a feasible way to improve the quality of nasturtium while not causing photosynthetic damage.

### 4.5. Plant Spacing and Effects of LAI on the RGR and LUE of Hydroponic Nasturtium

Under CL, the RGR for the same treatment were similar in the first 2 weeks of plant growth in both experiments (Table 2 and Table 3). It decreased in the third week in both experiments. The rate of decrease was higher, however, in Experiment 1 than in Experiment 2. Compared with the second week, the RGR decreased by 20% in Experiment 1, whereas it decreased by 11–13% in Experiment 2. This difference manifested as contrasting growth patterns of the leaves and aboveground biomass of nasturtium plants between the experiments: a liner growth pattern for the former (Figure 2) and an accelerated-growth pattern for the latter (Figure 4).

This difference could be attributed to the differences in plant spacing in the two experiments. In PFALs that are destined to pursue the efficiency in space (as well as light) utilization as high as possible, it is of vital importance to adjust plant spacing in the course of plant growth from the seedling (nursery) stage to the stage ready to harvest so as to ensure the best growth conditions for attaining the highest possible space utilization efficiency [31,32]. In the case of lettuce production, transplanting or respacing is usually carried out twice or three times, using the projected leaf area to determine the optimal timing and plant density [33,34]. 

In Experiment 1, all the growth parameters per m^2^ related to the aboveground biomass showed clear growth deceleration in the last growth stage (Supplementary Figure S2a–e), which indicated the plant density applied for week 3 was too high. The plant density in Experiment 2 was therefore reduced so that the deceleration of the growth parameters per m^2^ in the last growth stage was reduced or turned into growth acceleration (Supplementary Figure S3a–e). Especially, in week 3, the plant spacing with a lower plant density in Experiment 2 (5.6 against 11.1 plants m^−2^ in Experiment 1) accelerated the rate of leaf area expansion (239% against 78%), which resulted in a larger rate of increase in the LAI (69% against 19%). Both of our experiments revealed a clear linear relationship between the RGR and LAI (Supplementary Figure S1), which suggests the following causal chains: plant spacing influenced the LAI to further influence the RGR. By adjusting the LAI, therefore, the RGR could be fine-tuned. The critical threshold of the LAI is left for further study. 

Under the CL condition, the LUE was larger in Experiment 1 than in Experiment 2, regardless of the growth stage (Figure 3 and Figure 6). This was due most likely to the differences in plant densities between the two experiments—that is, the higher plant density at each stage led to a higher LUE. In the later growth stages from week 2 to week 3, the LUE declined significantly in Experiment 1 while it declined only slightly in Experiment 2, without any significant differences. This suggests that there may be an optimal plant density at which a stable LUE was maintained in the later stages in Experiment 1. In addition, at the seedling stage, the LUE in both experiments was relatively low, indicating that higher plant densities could be used in the early stage (week 1) for improving the LUE.

Beccafichi et al. [35], cultivating lettuce with different plant densities in open fields, reported that the LUE was not affected by the plant density, even though the maximum ground cover rate reached 100%. This may be because the plants in the field received a much higher DLI than the one used in the present experiment. There have been some studies, however, which reported that the LUE of Pak choi and cotton were significantly affected by the plant density [36,37,38]. This indicates that LUE could be optimized by keeping the plant density appropriately at a certain level.

Generally, with a higher plant density, the planting area tends to be covered by plants faster, and accordingly, it reaches the optimal LAI faster. As plants grow, the supply of assimilates increases with the increase in leaf area, and the optimal LUE can be reached earlier. The plant density should be adjusted when the yield does not continue to increase with the increasing LAI. Improvement of the energy use efficiency and space use efficiency in plant factories is of essential importance. It is an urgent research agenda to study the optimal balance among the LAI, RGR, and LUE under CL conditions.

## 5. Conclusions

Nasturtium is a functional herbal plant of great commercial prospects. In this study, we investigated the feasibility of continuous lighting (CL) on the cultivation of hydroponic nasturtium in a PFAL and examined the effects of CL with different daily light integrals (DLIs) on the growth and secondary metabolites of the plant.

Our experiments verified that the CL treatment (24 h per day lighting) against the 16 h per day lighting treatment at the same DLI of 17.3 mol m^−2^ d^−1^ enhanced the leaf and shoot dry weights, antioxidant capacity, and total phenolic contents in the leaves and light use efficiency (LUE) without causing any physiological stress on the plants and that, under the CL conditions for the DLI, which ranged from 17.3 to 34.6 mol m^−2^ d^−1^, the biomass of the leaves, shoots, and roots increased linearly as the DLI increased. 

We conclude that CL with no dark period is a feasible and beneficial technology for the mass and high-quality production of nasturtium in PFALs and that an increase in the DLI under CL increases the nasturtium biomass linearly up to the light intensity of 400 μmol m^−2^ s^−1^. The effects of a DLI higher than this level on nasturtium plants needs to be studied in the future. 

## Figures and Tables

**Table 1:** Two lighting treatments for the same level of the daily light integral (DLI) created by different combinations of light intensity and the light period in Experiment 1, and three lighting treatments for different levels of the DLI created by different light intensities under the condition of continuous lighting (CL) in Experiment 2.

Experiment	Treatment Symbols	Light Intensity (µmol m^−2^ s^−1^)	Light Period (h d^−1^)	DLI (mol m^−2^ d^−1^)
1	T16-300	300	16	17.3
	T24-200	200	24	
2	CL-200	200	24	17.3
	CL-300	300	24	25.9
	CL-400	400	24	34.6

Note: DLI (mol m^−2^ d^−1^) = PPFD (µmol m^−2^ s^−1^) × light period (h/d) × 3600 (s/h)/10^6^.

**Table 2:** The F_v_/F_max_, secondary metabolites, relative growth rate (RGR), and leaf area index (LAI) of the nasturtium plants grown under different light periods, but with the same daily light integral (DLI), at 7, 14, and 21 DAT in Experiment 1.

Parameter	Unit	DAT	Treatment ^z^				Difference (%) ^y^	T-Test ^x^ *p*-Value
			T16-300		T24-200			
F_v_/F_max_	(ratio)	14	0.828	a	0.836	a	1.0	0.234
		21	0.841	a	0.820	a	−2.6	0.020
Antioxidant capacity	(mg TE g^−1^ leaf FW)	14	1.654	a^2^*	2.316	a^1^*	40	5 × 10^−5^
		21	1.356	b	2.003	b	48	3 × 10^−4^
Total phenolic content	(mg GAE g^−1^ leaf FW)	14	2.041	a	2.251	a	10	0.040
		21	2.081	a	2.396	a	15	0.056
RGR	(g g^−1^ day)	7	0.20	a	0.24	a^3^*	19	0.015
		14	0.22	a^3^*	0.24	a	11	6 × 10^−4^
		21	0.18	b	0.19	b	5	0.038
LAI	(m^2^ m^−2^)	7	0.75	c	1.16	c	55	0.014
		14	4.64	b	5.83	b	26	0.007
		21	6.47	a^9^*	6.93	a^9^*	7	0.422

^z^ For the treatment, T16-300 represents the light period of 16 h per day, with a light intensity at 300 µmol m^−2^ s^−1^. T24-200 represents the light period of 24 h per day, with a light intensity at 200 µmol m^−2^ s^−1^. For each treatment and for each growth parameter, the numbers followed vertically (column-wise comparison) by different alphabet letters are statistically different, and the order of magnitude corresponds to the order of the alphabet, i.e., a > b > c. The signs, ^1^*, ^2^*, ^3^*, or ^9^*, put after the alphabet ‘a’ at the largest mean for each parameter and each treatment, indicate that the probability of the *t*-test (for the top three parameters) or of the ANOVA (the multiple comparison by Tukey for the RGR and LAI) is *p* < 0.05, *p* < 0.01, *p* < 0.001, or *p* < 1.0 × 10^−10^, respectively. ^y^ The rate of the difference between T24-20 and T16-300, i.e., ((T24–T16)/T16) *100. ^x^ Double-sided *t*-test with unequal variances for the mean difference between the two light period treatments (row-wise comparison). The *p*-values that are less than *p* = 0.05 are shown in bold letters.

**Table 3:** Secondary metabolites and relative growth rate (RGR) and leaf area index (LAI) of nasturtium plants grown under continuous lighting (CL) with three different daily light integrals (DLIs) at 7, 14, and 21 days after treatment (DAT) in Experiment 2.

Parameter	Unit	DAT	Treatment ^z^						Rate of Change (%)		ANOVA *p-*Value ^y^
			CL-200		CL-300		CL-400		CL300/ CL200	CL400/ CL300	
Antioxidant capacity	(mg TE/ g FW)	7	4.9	a^1^*	5.1	a	5.8	a^3^*	6	13	0.198
		14	3.6	ab	3.8	a	3.6	b	6	−5	0.952
		21	3.2	b	3.8	a	2.9	b	19	−24	0.137
Total phenolic content	(mg GA/ g FW)	7	3.1	a	3.2	a	3.5	a^3^*	2	9	0.256
		14	2.7	a	2.6	a	2.5	b	-3	−4	0.840
		21	2.5	a	2.8	a	2.5	b	12	−13	0.252
RGR of shoot DW	(g/g d)	7	0.26	a	0.28	a	0.32	a^1^*	8	13	0.027
		14	0.27	a^2^*	0.29	a^2^*	0.28	ab	8	−1	0.035
		21	0.24	b	0.25	b	0.26	b	4	5	6 × 10^−6^
LAI	(m^2^ m^−2^)	7	0.29	c	0.30	c	0.36	c	3	20	0.333
		14	1.81	b	2.14	b	1.91	b	18	−11	0.239
		21	3.06	a^14^*	3.25	a^10^*	4.12	a^12*^	6	27	0.001

^z^ For the treatment, CL-200, CL-300, and CL-400 represent the light period of 24 h per day with the light intensity of 200, 300, and 400 µmol m^−2^ s^−1^, respectively. For each treatment and for each parameter, numbers followed vertically (column-wise comparison) by different alphabet letters are statistically not different from each other (^1^* indicates the probability of ANOVA (the multiple comparison by Tukey) is ^1^* *p* < 0.05, ^2^* *p* < 0.01, 10* *p* < 1.0 × 10^−11^, ^12^* *p* < 1.0 × 10^−13^, and ^14^* *p* < 1.0 × 10^−15^), and the order of magnitude corresponds to the order of alphabet, i.e., a > b > c. ^y^ The *p*-values for the ANOVA of the multiple mean comparison among the three CL treatments by Tukey’s method applied to each row (row-wise comparison). The probabilities that are less than *p* = 0.05 are shown in bold letters.

## Data Availability

Not applicable.

## Supplementary Materials

The following are available online at https://www.mdpi.com/article/10.3390/plants10061203/s1: Table S1: Growth parameters of nasturtium plants grown under different daily light periods (Experiment 1) measured at 7, 14, and 21 days after treatment (DAT). Table S2: Growth parameters of nasturtium plants grown under continuous lighting (CL) with three different light intensities (Experiment 2) measured at 7, 14, and 21 days after treatment (DAT). Figure S1: Relationship between the relative growth rate (RGR) and the leaf area index (LAI) of nasturtium plants grown under two different light periods (Experiment 1) and under three different daily light integrals (DLI) (Experiment 2), Figure S2: Experiment 1: Growth parameters per m^2^ by growth stage, obtained by multiplying the plant density (no. of plant/m^2^) by the per-plant data in Figure 2 for their respective growth stages. Figure S3: Experiment 2: Growth parameters per m^2^ by growth stage, obtained by multiplying the plant density (no. of plant/m^2^) by the per-plant data in Figure 4 for their respective growth stages.

## References

[1] Xu W., Lu N., Kikuchi M., Takagaki M. (2021). Effects of Node Position and Electric Conductivity of Nutrient Solution on Adventitious Rooting of Nasturtium (*Tropaeolum majus* L.) Cuttings. Agronomy.

[2] Zhang X., He D., Niu G., Yan Z., Song J. (2018). Effects of environment lighting on the growth, photosynthesis, and quality of hydroponic lettuce in a plant factory. Int. J. Agric. Biol. Eng..

[3] Gao W., He D., Ji F., Zhang S., Zheng J. (2020). Effects of daily light integral and LED spectrum on growth and nutritional quality of hydroponic spinach. Agronomy.

[4] Yan Z., He D., Niu G., Zhou Q., Qu Y. (2019). Growth, nutritional quality, and energy use efficiency of hydroponic lettuce as influenced by daily light integrals exposed to white versus white plus red light-emitting diodes. HortScience.

[5] Yang L., Wen K.S., Ruan X., Zhao Y.X., Wei F., Wang Q. (2018). Response of plant secondary metabolites to environmental factors. Molecules.

[6] Nguyen D.T.P., Lu N., Kagawa N., Takagaki M. (2019). Optimization of photosynthetic photon flux density and root-zone temperature for enhancing secondary metabolite accumulation and production of coriander in plant factory. Agronomy.

[7] Sysoeva M., Markovskaya E., Shibaeva T. (2010). Plants under Continuous Light: A Review. Plant Stress.

[8] Mao H., Hang T., Zhang X., Lu N. (2019). Both Multi-Segment Light Intensity and Extended Photoperiod Lighting Strategies, with the Same Daily Light Integral, Promoted Lactuca sativa L. Growth and photosynthesis. Agronomy.

[9] Weaver G., van Iersel M.W. (2020). Longer photoperiods with adaptive lighting control can improve growth of greenhouse-grown ‘Little Gem’ lettuce (*Lactuca sativa*). HortScience.

[10] Palmer S., van Iersel M.W. (2020). Increasing growth of lettuce and mizuna under sole-source LED lighting using longer photoperiods with the same daily light integral. Agronomy.

[11] Kitaya Y., Niu G., Kozai T., Ohashi M. (1998). Photosynthetic photon flux, photoperiod, and CO2 concentration affect growth and morphology of lettuce plug transplants. HortScience.

[12] Ohyama K., Manabe K., Omura Y. (2005). Potential Use of a 24-Hour Photoperiod (Continuous Light) with Alternating Air Temperature for Production of Tomato Plug Transplants in a Closed System. HortScience.

[13] Ohyama K., Omura Y., Kozai T. (2005). Effects of air temperature regimes on physiological disorders and floral development of tomato seedlings grown under continuous light. HortScience.

[14] Velez-Ramirez A.I., Van Ieperen W., Vreugdenhil D., Millenaar F.F. (2011). Plants under continuous light. Trends Plant Sci..

[15] Shimomura M., Yoshida H., Fujiuchi N., Ariizumi T., Ezura H., Fukuda N. (2020). Continuous blue lighting and elevated carbon dioxide concentration rapidly increase chlorogenic acid content in young lettuce plants. Sci. Hortic..

[16] Zha L., Zhang Y., Liu W. (2019). Dynamic Responses of Ascorbate Pool and Metabolism in Lettuce to Long-term Continuous Light Provided by Red and Blue LEDs. Environ. Exp. Bot..

[17] Avio L., Sbrana C., Giovannetti M., Frassinetti S. (2017). Arbuscular mycorrhizal fungi affect total phenolics content and antioxidant activity in leaves of oak leaf lettuce varieties. Sci. Hortic..

[18] Kitayama M., Nguyen D.T.P., Lu N., Takagaki M. (2019). Effect of light quality on physiological disorder, growth, and secondary metabolite content of water spinach (*Ipomoea aquatica* forsk) cultivated in a closed-type plant production system. Hortic. Sci. Technol..

[19] Kozai T., Niu G., Kozai T., Niu G., Takagaki M. (2016). Role of the plant factory with artificial lighting (PFAL) in urban areas. Plant Factory–An Indoor Vertical Farming System for Efficient Quality Food Production.

[20] Gauslaa Y., Solhaug K.A. (2000). High-light-intensity damage to the foliose lichen *Lobaria pulmonaria* within a natural forest: The applicability of chlorophyll fluorescence methods. Lichenologist.

[21] Li S., Yang W., Yang T., Chen Y., Ni W. (2015). Effects of Cadmium Stress on Leaf Chlorophyll Fluorescence and Photosynthesis of *Elsholtzia argyi*-A Cadmium Accumulating Plant. Int. J. Phytoremediation.

[22] Camejo D., Nicolás E., Torres W., Alarcón J.J. (2010). Differential heat-induced changes in the CO2 assimilation rate and electron transport in tomato (*Lycopersicon esculentum* Mill.). J. Hortic. Sci. Biotechnol..

[23] Velez-ramirez A.I., Van Ieperen W., Vreugdenhil D., Van Poppel P.M.J.A., Heuvelink E., Millenaar F.F. (2014). A single locus confers tolerance to continuous light and allows substantial yield increase in tomato. Nat. Commun..

[24] Minh D., Chun C. (2020). Growth and leaf injury in tomato plants under continuous light at di ff erent settings of constant and diurnally varied photosynthetic photon fl ux densities. Sci. Hortic..

[25] Kelly N., Choe D., Meng Q., Runkle E.S. (2020). Promotion of lettuce growth under an increasing daily light integral depends on the combination of the photosynthetic photon flux density and photoperiod. Sci. Hortic..

[26] Dou H., Niu G., Gu M., Masabni J.G. (2018). Responses of sweet basil to different daily light integrals in photosynthesis, morphology, yield, and nutritional quality. HortScience.

[27] Maxwell K., Johnson G.N. (2000). Chlorophyll fluorescence-A practical guide. J. Exp. Bot..

[28] Zha L., Liu W., Zhang Y., Zhou C., Shao M. (2019). Morphological and Physiological Stress Responses of Lettuce to Different Intensities of Continuous Light. Front. Plant Sci..

[29] Carvalho I.S., Cavaco T., Carvalho L.M., Duque P. (2010). Effect of photoperiod on flavonoid pathway activity in sweet potato (*Ipomoea batatas* (L.) Lam.) leaves. Food Chem..

[30] Pennisi G., Pistillo A., Orsini F., Cellini A., Spinelli F., Nicola S., Fernandez J.A., Crepaldi A., Gianquinto G., Marcelis L.F.M. (2020). Optimal light intensity for sustainable water and energy use in indoor cultivation of lettuce and basil under red and blue LEDs. Sci. Hortic..

[31] Ioslovich I., Gutman P.O. (2000). Optimal control of crop spacing in a plant factory. Automatica.

[32] Lu N., Shimamura S. (2018). Protocols, Issues and Potential Improvements of Current Cultivation Systems. Smart Plant Fact..

[33] Nunomura O., Kozai T., Shinozaki K., Oshio T., Kozai T., Niu G., Takagaki M. (2016). Seeding, seedling production and transplanting. Plant Factory–An Indoor Vertical Farming System for Efficient Quality Food Production.

[34] Kozai T., Sakaguchi S., Akiyama T., Yamada K., Ohshima K., Kozai T., Niu G., Takagaki M. (2016). Design and Management of PFAL. Plant Factory–An Indoor Vertical Farming System for Efficient Quality Food Production.

[35] Beccafichi C., Benincasa P., Guiducci M., Tei F. (2003). Effect of crop density on growth and light interception in greenhouse lettuce. Acta Hortic..

[36] Cho Y.Y., Lee J.H., Shin J.H., Son J.E. (2015). Development of an expolinear growth model for pak-choi using the radiation integral and planting density. Hortic. Environ. Biotechnol..

[37] Cho Y.Y., Son J.E. (2005). Effects of planting density on growth and yield of hydroponically-grown pak-choi (Brassica campestris ssp. chinensis). Hortic. Environ. Biotechnol..

[38] Chapepa B., Mudada N., Mapuranga R. (2020). The impact of plant density and spatial arrangement on light interception on cotton crop and seed cotton yield: An overview. J. Cott. Res..

