# Supplementary material for: Continuous Lighting and High Daily Light Integral Enhance Yield and Quality of Mass-Produced Nasturtium (Tropaeolum majus L.) in Plant Factories
Source: Plants (Basel). 2021 Jun 12;10(6):1203. doi: 10.3390/plants10061203 (PMC8231634; doi:10.3390/plants10061203)
Supplement: Supplementary file 1 [file plants-10-01203-s001.zip › plants-1236794-supplementary.pdf]

Continuous lighting and high daily light integral enhance yield and quality of mass-produced nasturtium (*Tropaeolum majus* L.) in plant factories—Supplementary

Table S1. Growth parameters of nasturtium plants grown under different daily light periods (Experiment 1) measured at 7, 14, and 21 days after treatment (DAT). The plant densities for 7, 14, and 21 DAT are 21.5, 16.7, and 11.1 plants/m^2, respectively.

Growth parameter	Unit	DAT	Treatment ^{z} T16-300		Treatment ^{z} T24-200		Difference (%) ^{y}	*t*-test ^{x} *p*-value
Leaf FW	(g/plant)	7	8.1	(0.2)	11.1	(0.9)	37	**0.023**
		14	40.8	(1.3)	50.2	(2.2)	23	**0.006**
		21	69.9	(4.1)	77.5	(4.3)	11	0.236
Leaf DW	(g/plant)	7	1.2	(0.03)	1.7	(0.15)	41	**0.020**
		14	4.2	(0.20)	5.4	(0.23)	30	**0.002**
		21	7.4	(0.33)	8.8	(0.46)	18	**0.044**
Leaf no.	(no./plant)	7	39	(2)	47	(3)	23	**0.028**
		14	100	(2)	116	(2)	17	**0.001**
		21	174	(8)	188	(12)	8	0.354
Leaf area	(cm^2/plant)	7	348	(15)	541	(53)	55	**0.014**
		14	2786	(120)	3500	(169)	26	**0.007**
		21	5824	(358)	6234	(333)	7	0.422
Shoot FW	(g/plant)	7	14	(1)	20	(2)	43	**0.023**
		14	130	(5)	179	(6)	38	**$2x10^{-4}$**
		21	314	(23)	362	(8)	15	0.136
Shoot DW	(g/plant)	7	1.6	(0.06)	2.1	(0.15)	31	**0.020**
		14	7.8	(0.38)	11.1	(0.57)	42	**0.001**
		21	18.2	(1.07)	21.8	(1.14)	20	**0.041**
Root FW	(g/plant)	7	6.2	(0.3)	9.2	(0.9)	48	**0.023**
		14	27.5	(1.3)	35.8	(1.3)	30	**0.001**
		21	46.3	(2.5)	52.0	(2.9)	12	0.166
Root DW	(g/plant)	7	0.26	(0.01)	0.37	(0.03)	42	**0.009**
		14	0.86	(0.02)	1.11	(0.02)	29	**$2x10^{-5}$**
		21	7.97	(0.17)	8.11	(0.16)	2	0.568

^{z} For treatment, T16-300 represents the light period of 16 h per day with a light intensity at 300 μmol m^{-2} s^{-1}. T24-200 represents the light period of 24 h per day with a light intensity at 200 μmol m^{-2} s^{-1}. Numbers in parentheses are standard errors.

^{y} The rate of differences between T24-20 and T16-300, i.e., ((T24 - T16)/T16) *100. The sign 'ns' means that the mean difference was not statistically significant.

^{x} Double-sided *t*-test with unequal variances for the mean difference between the two treatments. The *p*-values that are less than $p = 0.05$ are shown in bold letters.

Table S2. Growth parameters of nasturtium plants grown under continuous lighting (CL) with three different light intensities (Experiment 2) measured at 7, 14, and 21 days after treatment (DAT). The plant densities for 7, 14, and 21 DAT are 16.7, 11.1, and 5.6 plants/m^2, respectively.

Growth parameter	Unit	DAT	Treatment [z]						Rate of change (%)		ANOVA p-value [y]
			CL-200		CL-300		CL-400		CL300/ CL200	CL400/ CL300	
Leaf FW	(g/plant)	7	3.9	a	4.3	a	5.3	a	12	21	0.146
		14	29	b	37	a	35	ab	29	-7	**0.049**
		21	86	b	100	b	137	a	16	38	**0.003**
Leaf DW	(g/plant)	7	0.57	b	0.71	ab	0.95	a	23	34	**0.023**
		14	3.3	b	4.7	a	4.5	a	40	-4	**0.009**
		21	10	b	12	b	15	a	20	27	**3x10^{-4}**
Leaf no.	(no./plant)	7	15	a	18	a	20	a	14	11	0.091
		14	50	a	54	a	56	a	7	4	0.399
		21	167	a	188	a	203	a	12	8	0.131
Leaf area	(cm^2/plant)	7	173	a	181	a	217	a	4	20	0.333
		14	1627	a	1925	a	1716	a	18	-11	0.239
		21	5509	b	5851	b	7411	a	6	27	**0.001**
Shoot FW	(g/plant)	7	6.4	a	6.9	a	8.3	a	8	20	0.203
		14	68	a	87	a	81	a	28	-7	0.141
		21	291	b	346	b	480	a	19	39	**2x10^{-6}**
Shoot DW	(g/plant)	7	0.80	b	0.94	ab	1.26	a	17	34	**0.010**
		14	5.4	b	7.3	a	7.0	ab	36	-3	**0.039**
		21	20	b	24	b	32	a	24	32	**9x10^{-6}**
Root FW	(g/plant)	7	3.3	a	3.8	a	4.6	a	13	21	0.242
		14	21	a	27	a	26	a	24	-3	0.213
		21	59	b	80	b	111	a	36	39	**3x10^{-5}**
Root DW	(g/plant)	7	0.12	a	0.14	a	0.19	a	12	41	0.056
		14	0.69	a	0.86	a	0.83	a	25	-4	0.200
		21	1.9	c	2.7	b	3.3	a	40	25	**1x10^{-5}**

[z] For treatment, CL-200, CL-300, and CL-400 represent the light period of 24 h per day with the light intensities of 200, 300, and 400 μmol m^{-2} s^{-1}, respectively. For each row, the figures followed horizontally by the same alphabet letter are not statistically different from each other at the significance level of $p < 0.05$. The order of alphabet letters, a, b, c, ..., corresponds to the order of the magnitudes of the respective parameters.

[y] The p-values for ANOVA (the multiple mean comparison by Tukey's method) are the probabilities, and those that are less than $p = 0.05$ are shown in bold letters.

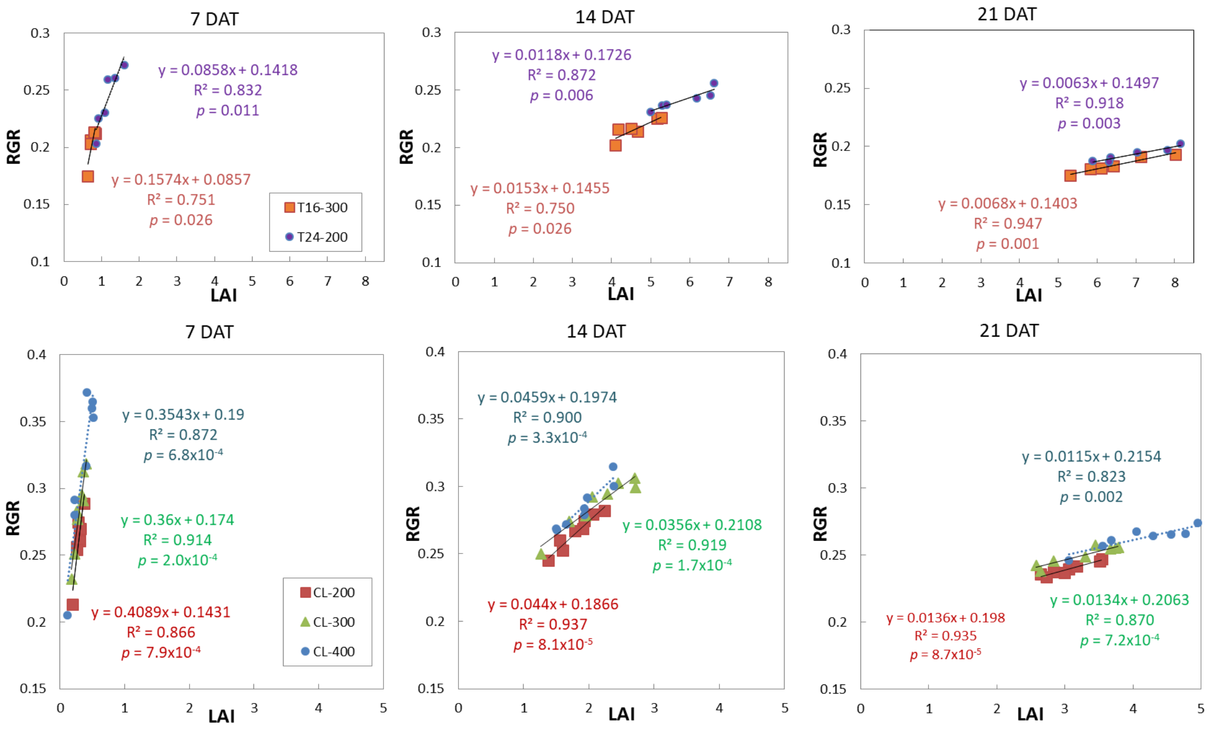

Figure S1. Relationship between the relative growth rate (RGR) and the leaf area index (LAI) of nasturtium plants grown under two different light periods (Experiment 1) and under three different daily light integrals (DLI) (Experiment 2). DAT = days after treatment. Each regression equation is of the following model: y = a X + b, where y = RGR, x = LAI, and a (slope coefficient) and b (intercept) are the coefficients to be estimated. '*p*' is the provability of the F-value of the regression estimation, with $n = 6$ for each equation in the top three charts and $n = 8$ for the bottom three charts.

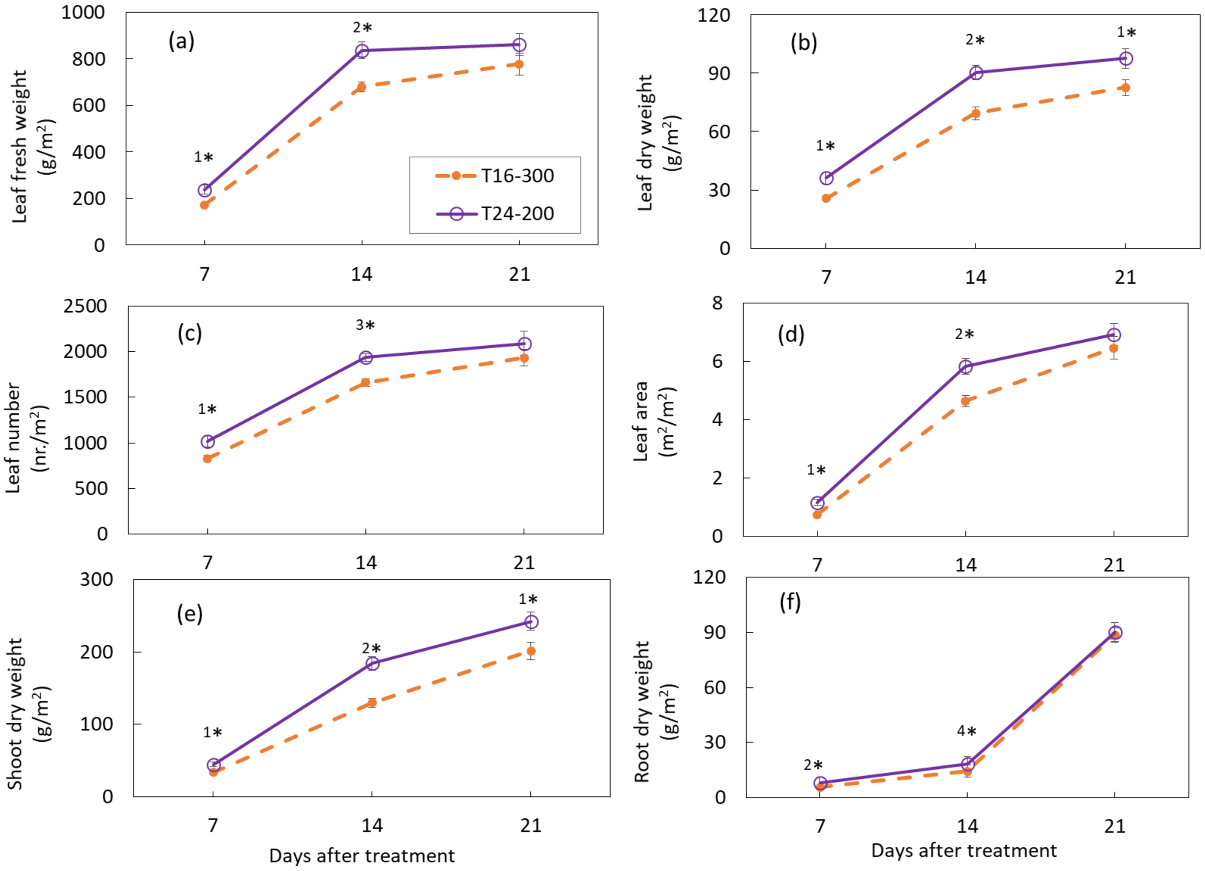

Figure S2. Experiment 1: Growth parameters per m^2 by growth stage, obtained by multiplying the plant density (no. of plant/m^2) to the per-plant data in Figure. 2 for their respective growth stages. The plant densities for 7, 14, and 21 DAT were 21.5, 16.7, and 11.1 plants/m^2, respectively. Values are the means ± SE (n = 6). Asterisks indicate significant differences between treatments (^{1*}, $p < 0.05$; ^{2*}, $p < 0.01$; ^{3*}, $p < 0.001$), determined by the t-test.

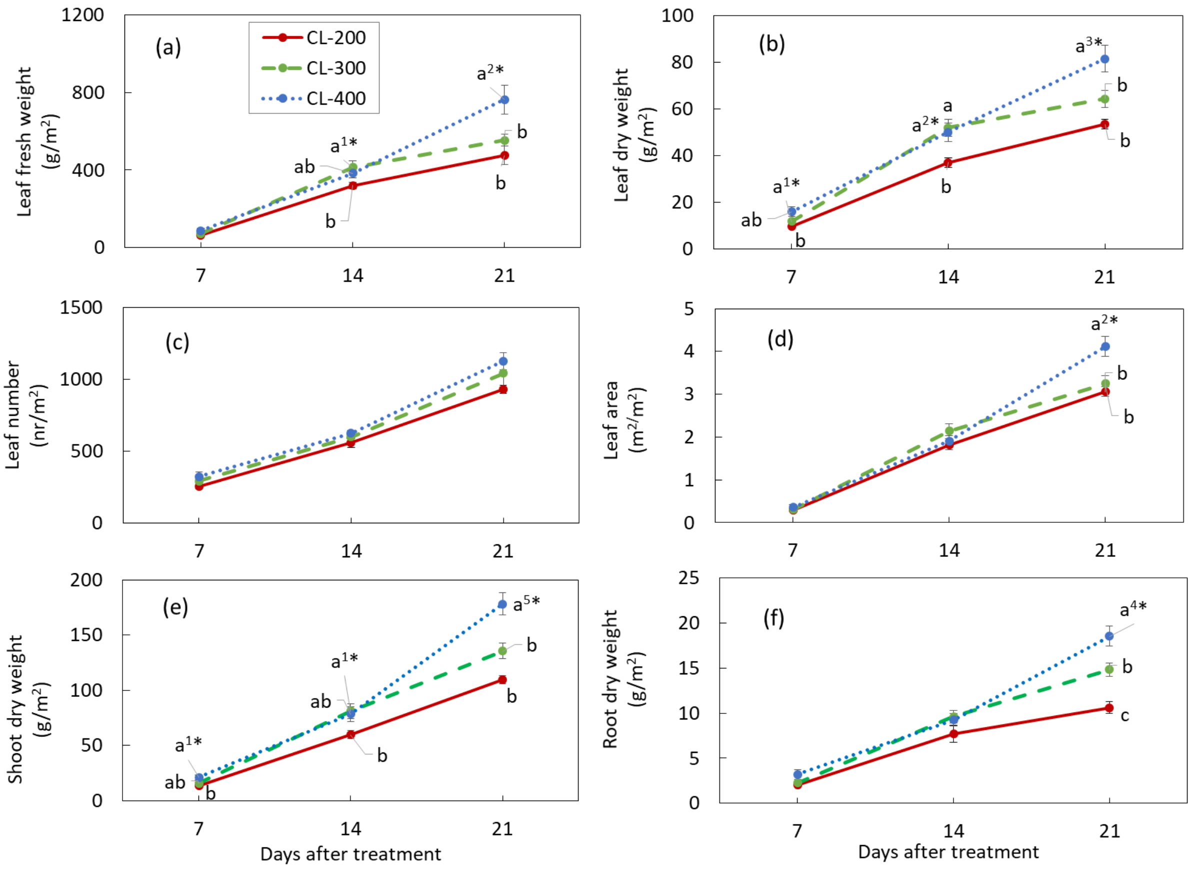

Figure S3. Experiment 2: Growth parameters per m² by growth stage, obtained by multiplying the plant density (no. of plant/m²) to the per-plant data in Figure. 4 for their respective growth stages. The plant densities for 7, 14, and 21 DAT are 16.7, 11.1, and 5.6 plants/m², respectively. Values are the means ± SE (n = 8). Different alphabet letters indicate significant differences between the treatments, the signs, ^{1*}, ^{2*}, ^{3*}, ^{4*}, or ^{5*} put after the alphabet 'a' at the largest mean for each parameter and each 'days after treatment', indicating that the probability of the ANOVA (the multiple mean comparison by Tukey) was $p < 0.05$, $p < 0.01$, $p < 0.001$, $p <$ 1.0E-04, or $p <$ 1.0E-05, respectively.
